# Supplementary material for: Systematic functional interrogation of human pseudogenes using CRISPRi
Source: Genome Biol. 2021 Aug 23;22:240. doi: 10.1186/s13059-021-02464-2 (PMC8381491; doi:10.1186/s13059-021-02464-2)

Supplementary Information

Supplementary Figure 1. (**A**) The histogram showing the distribution of the number of sgRNAs per pseudogene in the CRISPRi library. (**B**) The scatter plots showing the correlation of sgRNA abundance between three replicates on day 0 and day 21. (**C**) The histograms showing the sgRNA abundance distribution for pseudogenes, parent genes and positive/negative controls on day 0 and day 21. The histograms showing the **(D)** sgRNA-level and (**E**) gene-level distribution of log_2(Fold-Change) between day 21 and day 0 for parent genes, pseudogenes, positive controls and essential parent genes (Ess parent) identified from previous CRISPR-Cas9 knockout screens. (**F**) The histograms showing the distribution of the number of Cas-OFFinder-predicted genomic off-target sites for sgRNAs targeting pseudogenes and parent genes. (**G**) The boxplots showing the number of Cas-OFFinder-predicted genomic off-target sites for sgRNAs targeting pseudogenes/parent genes that are screen hits and the ones that are not.

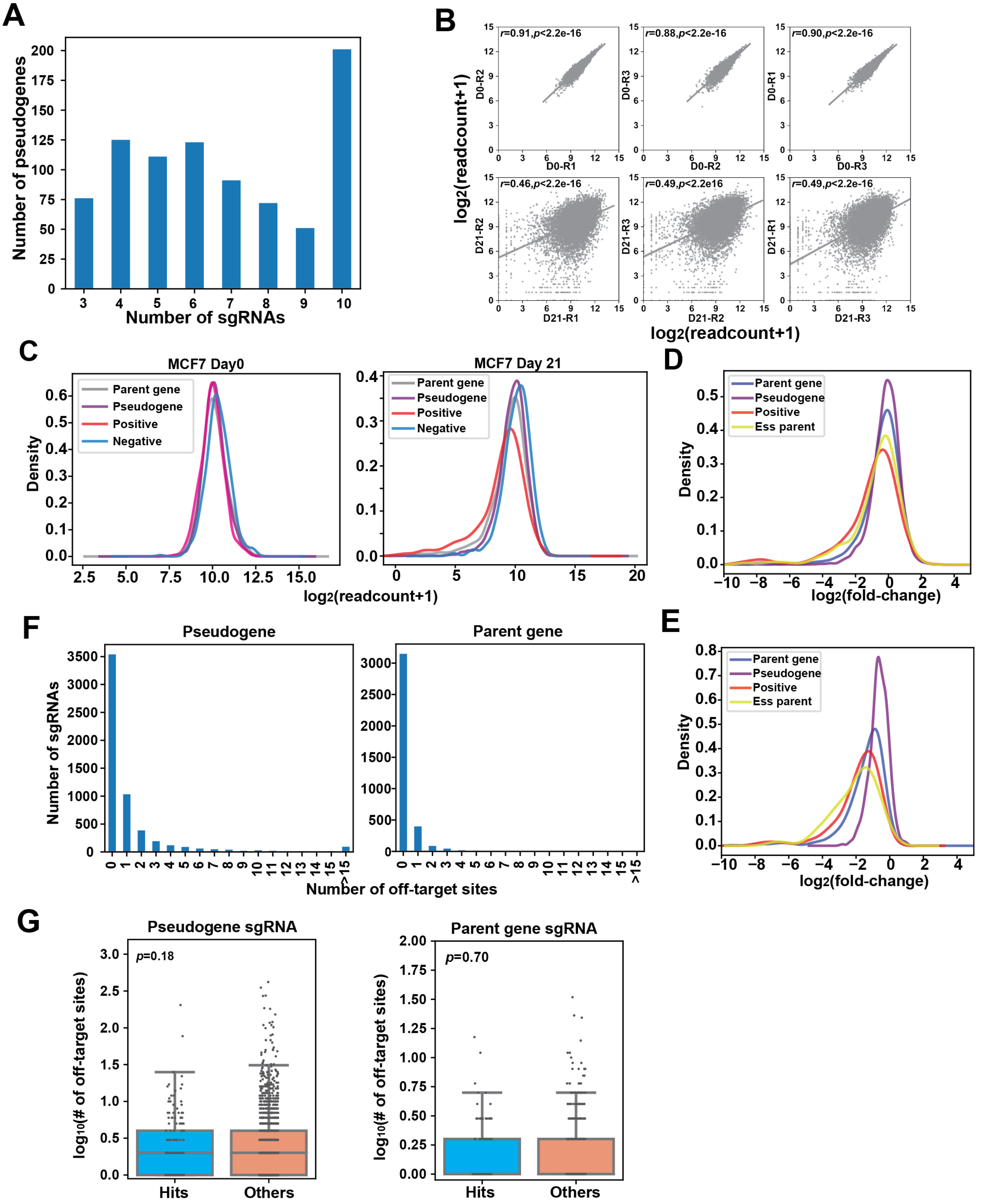
A
Number of pseudogenes
Number of sgRNAs
B
r=0.91,p<2.2e-16
r=0.88,p<2.2e-16
r=0.90,p<2.2e-16
r=0.46,p<2.2e-16
r=0.49,p<2.2e16
r=0.49,p<2.2e-16
D0-R2
D0-R3
D0-R1
D21-R2
D21-R3
D21-R1
D0-R1
D0-R2
D0-R3
D21-R1
D21-R2
D21-R3
log2(readcount+1)
log2(readcount+1)
C
MCF7 Day0
MCF7 Day 21
Parent gene
Pseudogene
Positive
Negative
Density
log2(readcount+1)
D
Parent gene
Pseudogene
Positive
Ess parent
Density
log2(fold-change)
F
Pseudogene
Parent gene
Number of sgRNAs
Number of off-target sites
E
Parent gene
Pseudogene
Positive
Ess parent
Density
log2(fold-change)
G
Pseudogene sgRNA
Parent gene sgRNA
p=0.18
p=0.70
log10(# of off-target sites)
log10(# of off-target sites)
Hits
Others
Hits
Others

Supplementary Figure 2. **(A)** QRT-PCR was performed to determine the sgRNA-mediated knockdown efficiency for pseudogene MGAT4EP, DDX12P, PRELID1P1 and TUBBP5 in MCF7-dCas9 cells. **(B)** The representative pictures of clonogenic growth and the bar graph quantifying the colonies formed by the MCF7-dCas9 cells transduced with a negative control of sg-AAVS1/sg-nAAVS1 or individual gene-specific sgRNAs for MGAT4EP, DDX12P, PRELID1P1 and TUBBP5, after cells were cultured for two weeks. **(C)** The growth of the MCF7-dCas9 cells transduced with a negative control of sg-AAVS1/sg-nAAVS1, or individual gene-specific sgRNAs for MGAT4EP, DDX12P, PRELID1P1 and TUBBP5, was monitored (OD450 absorbance for WST-8 formazen) every 24 hours with CCK-8 assay for 96 hours. **(D)** The growth of the MCF7-dCas9 cells co-transduced with sg-AAVS1 and an empty vector control (EV), or a gene-specific sgRNA together with an EV/a cDNA overexpression vector (OE), was monitored every 24 hours with CCK-8 assay for 96 hours. **(E)** The representative pictures of clonogenic growth and the bar graph quantifying the colonies formed by the MCF7-dCas9 cells co-transduced with sg-AAVS1 and EV, or a gene-specific sgRNA together with an EV/OE, after cells were cultured for two weeks. All data are shown as mean±SD, n=3. The Student's *t*-test was used to assess the statistical significance of difference in mean between two experimental groups (*$p<0.05$; **$p<0.01$; ns: not significant, $p\geq0.05$).

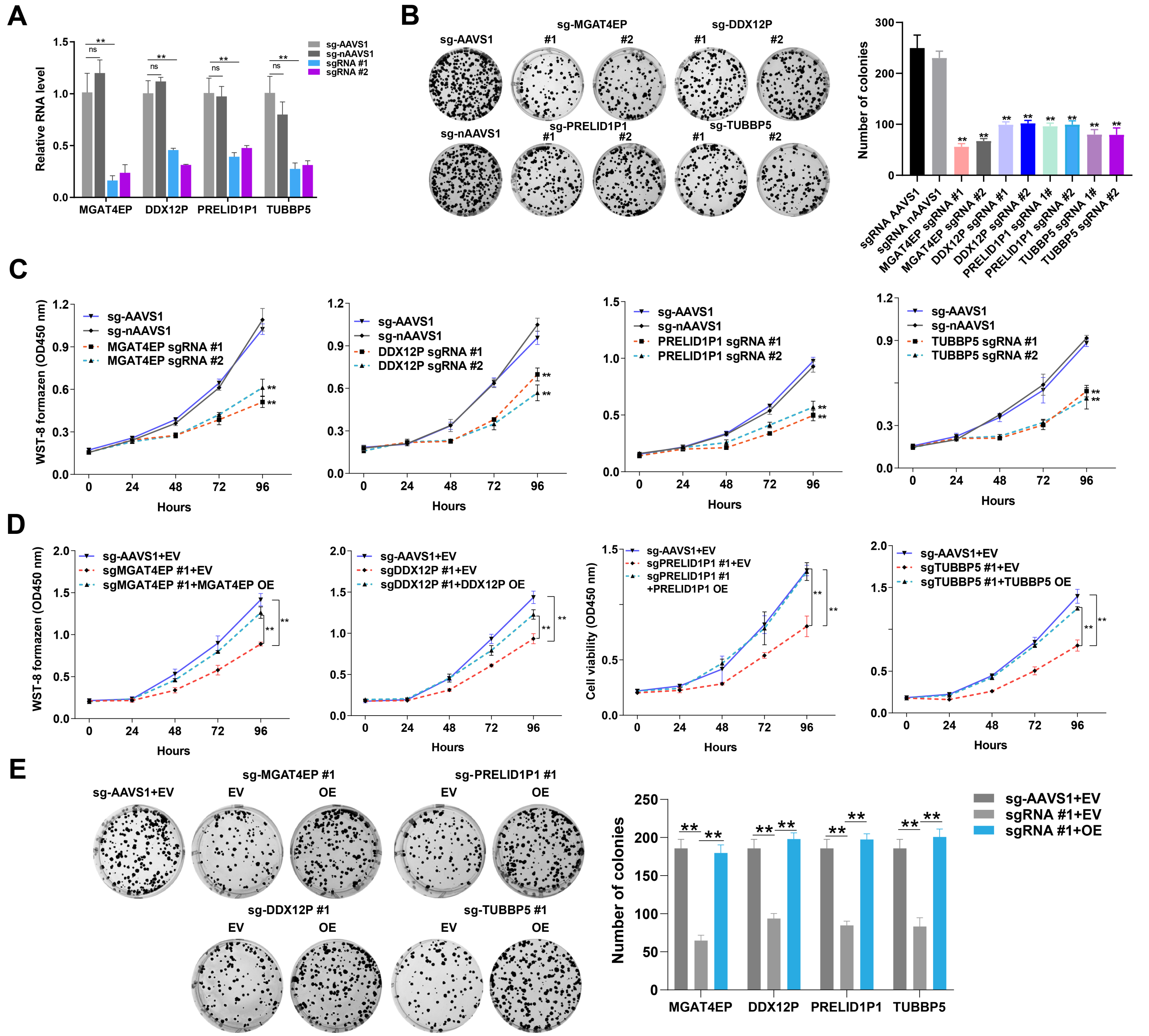

Supplementary Figure 3. **(A)** ChIP-qPCR was performed with anti-Pol II or anti-IgG antibody to determine the binding of pol II to the promoters of pseudogene PRELID1P1 and its parent gene PRELID1 in MCF7-dCas9 cells that were transduced with sg-NT or the first PRELID1P1-targeting sgRNA. **(B)** The boxplot showing the expression of MGAT4EP in breast cancer and normal breast tissue based on TCGA RNA-seq data. **(C)** The violin plot showing the expression of MGAT4EP in different normal tissues based on GTEx RNA-seq data. **(D)** An agarose gel stained with ethidium bromide revealing that the size of 5' and 3' RACE product was consistent with the Refseq-annotated MGAT4EP transcript (NR_038135.2). **(E)** An *in vitro* translation assay was performed to determine whether the MGAT4EP could encode proteins/micropeptides in MCF7 cells. Luciferase (Luc) was used as a positive control and no template condition was used as a negative control (NC). R1 and R2 represent the two replicates for MGAT4EP translation. **(F)** QRT-PCR analysis of MGAT4EP expression in MCF7 cells that were treated with negative control non-targeting siRNA (si-NC) or MGAT4EP-targeting siRNA. **(G)** The growth of MCF7 and T47D cells treated with si-NC or MGAT4EP-targeting siRNA was monitored (OD450 absorbance for WST-8 formazen) every 24 hours with CCK-8 assay for 96 hours. **(H)** Western blot for measuring β-tubulin and histone H3 protein expression in the cytoplasmic and nuclear fraction of MCF7 and T47D cells. All data are shown as mean±SD, n=3. The Student's *t*-test was used to assess the statistical significance of difference in mean between two experimental groups (**p*<0.05; ***p*<0.01; ns: not significant, *p*≥0.05).

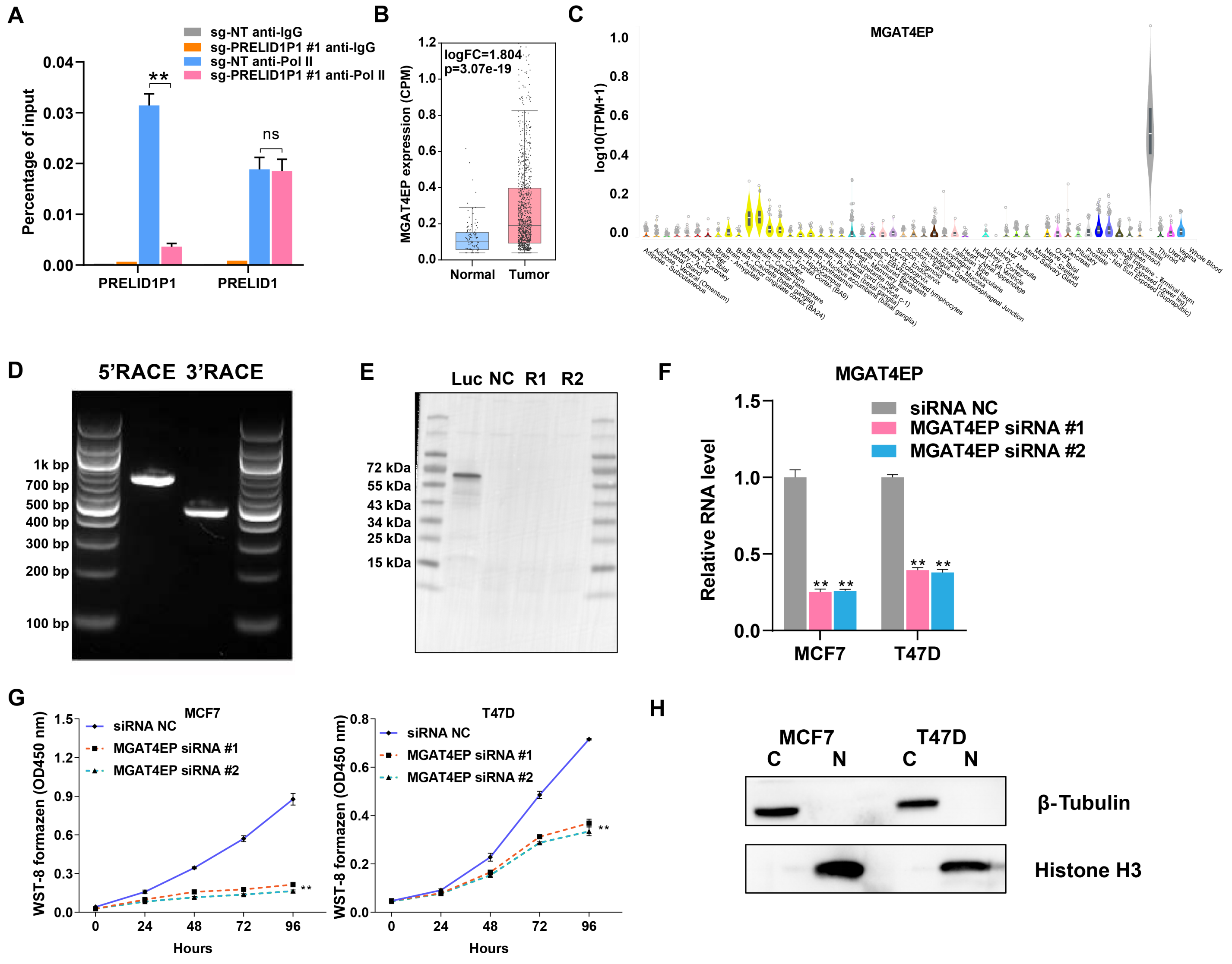

Supplementary Figure 4. **(A)** The table summarizing the information about eight annotated transcription factors/epigenetic regulators that were specifically enriched by MAGAT4EP RNA pull-down compared with antisense (AS) control and that showed significant up-regulation in luminal A breast cancer compared with normal breast tissues. **(B)** The heatmap showing the significantly up-/down-regulated protein-coding genes between MCF7-dCas9 cells transduced with sg-NT and those transduced with MGAT4EP-targeting sgRNA. **(C)** The boxplots showing the mRNA expression of FOXA1 and FOXM1 in luminal A breast cancer and normal breast tissue based on TCGA RNA-seq data. (**D**) QRT-PCR analysis of FOXM1 mRNA expression and (**E**) western blot for measuring FOXM1 protein expression in MCF7-dCas9 cells that were transduced with sg-NT or MGAT4EP-targeting sgRNAs. (**F**) Western blot for measuring FOXA1 protein expression in MCF7 and T47D cells that were treated with si-NC or MGAT4EP-targeting siRNA. (**G**) The representative pictures of clonogenic growth and the bar graph quantifying the colonies formed by MCF7 cells transduced with sh-NC or FOXM1-targeting shRNA, after cells were cultured for two weeks. All data are shown as mean±SD, n=3. The Student's *t*-test was used to assess the statistical significance of difference in mean between two experimental groups (**p*<0.05; ***p*<0.01; ns: not significant, *p*≥0.05).

A

Gene	MWT(kDa)	Unique AS	MGAT4EP_Unique	luminalA_log2FC	luminalA_padj
ASF1B	22.42	0	2	2.497658	1.8E-154
CBX4	61.33	0	2	1.648218	6.78E-97
CBX8	43.37	0	4	1.304425	2.51E-55
FOXA1	49.12	0	2	2.231704	9.54E-122
ESRP1	75.54	0	2	1.390098	5.61E-52
INTS7	106.77	0	7	1.151371	5.1E-102
MSI2	35.17	0	2	1.173382	3.13E-66
NUDT16L1	23.32	0	5	1.364836	1.65E-95

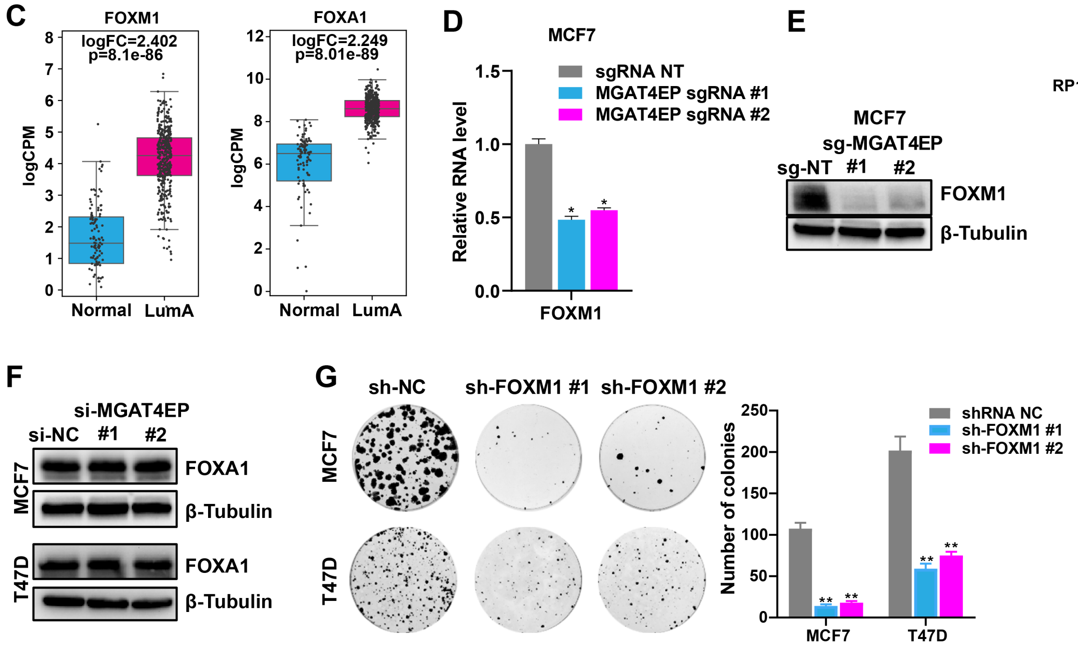

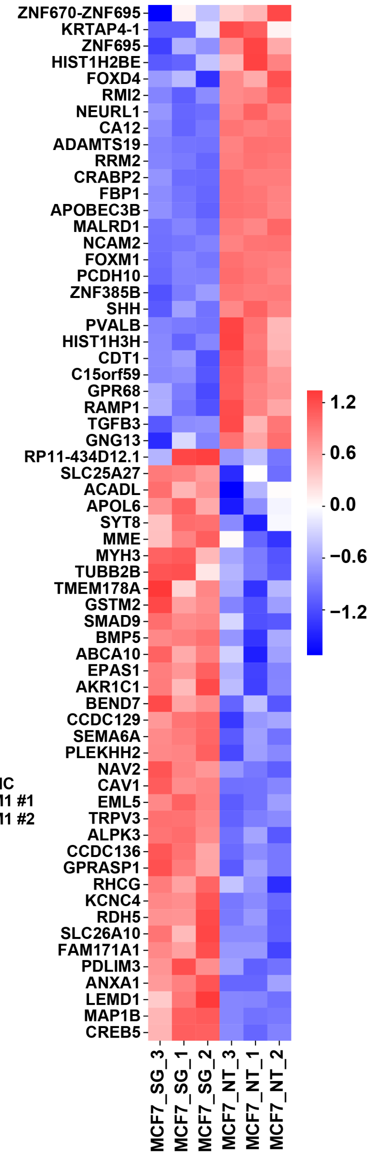

Supplementary Figure 5. The source data of gel pictures in Figure 4B, C, D and E.

Figure 4B

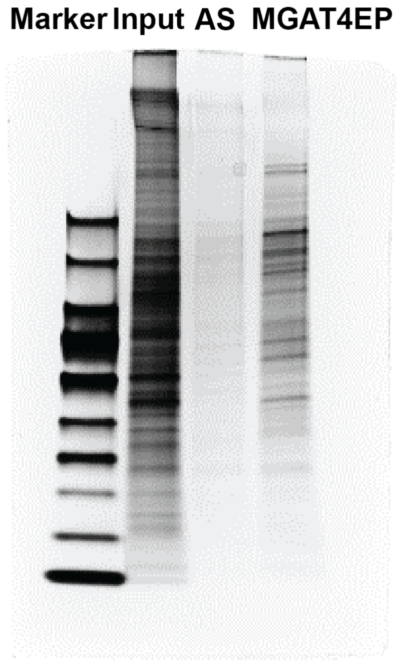

Figure 4C

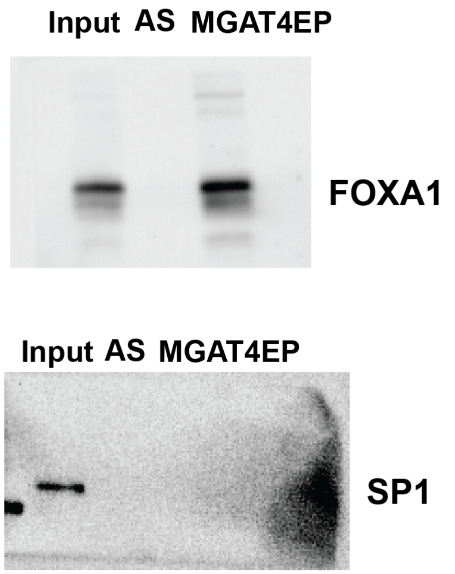

Figure 4D

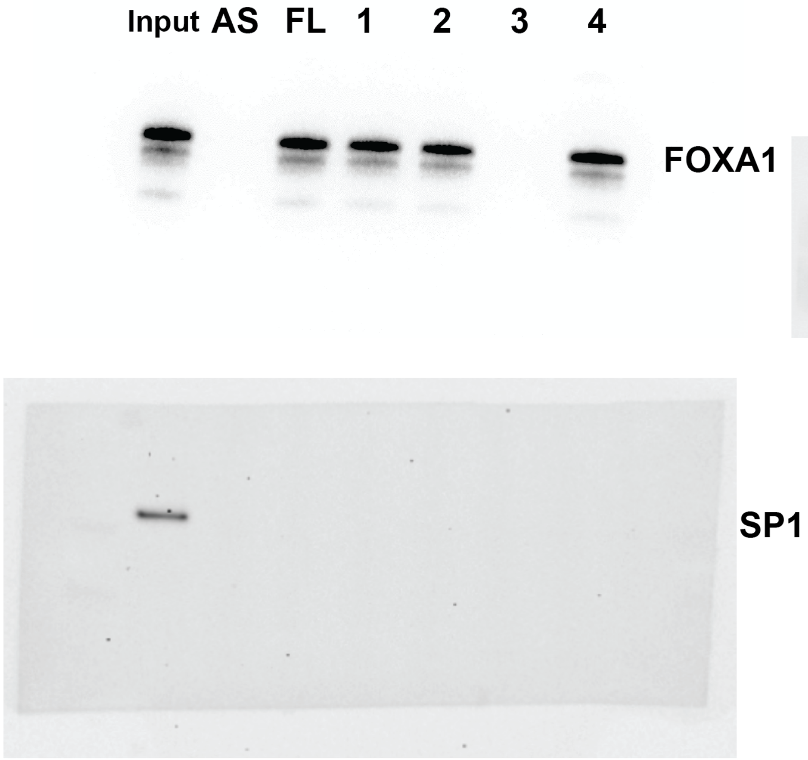

Figure 4E

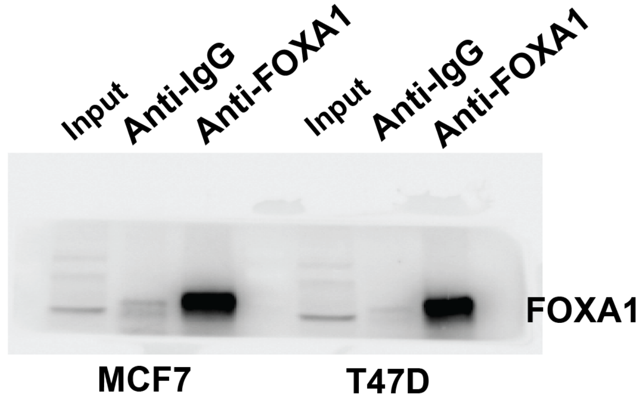

Supplementary Figure 6. The source data of gel pictures in Figure 5C, E and H.

Figure 5C

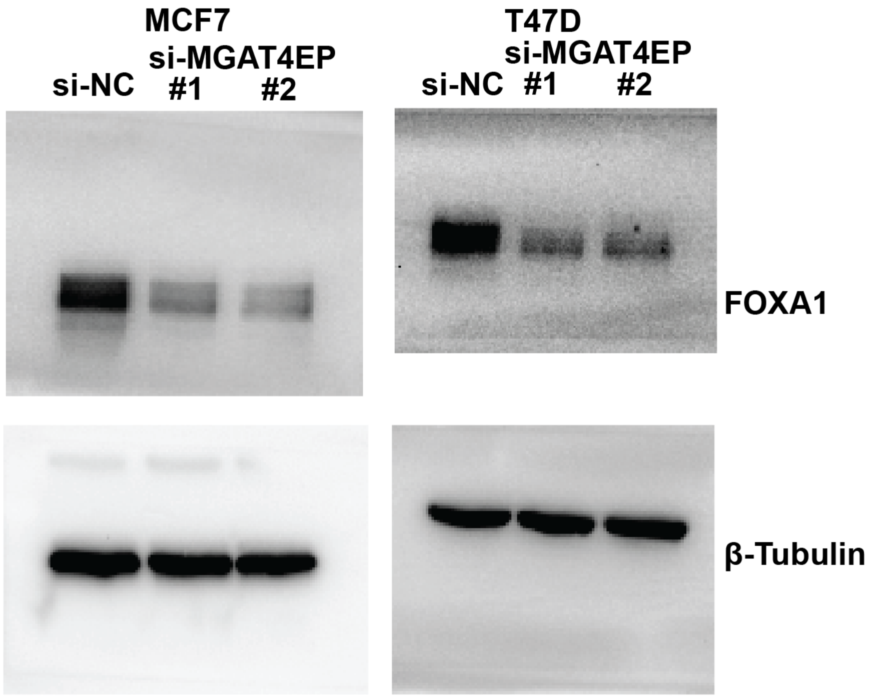

Figure 5E

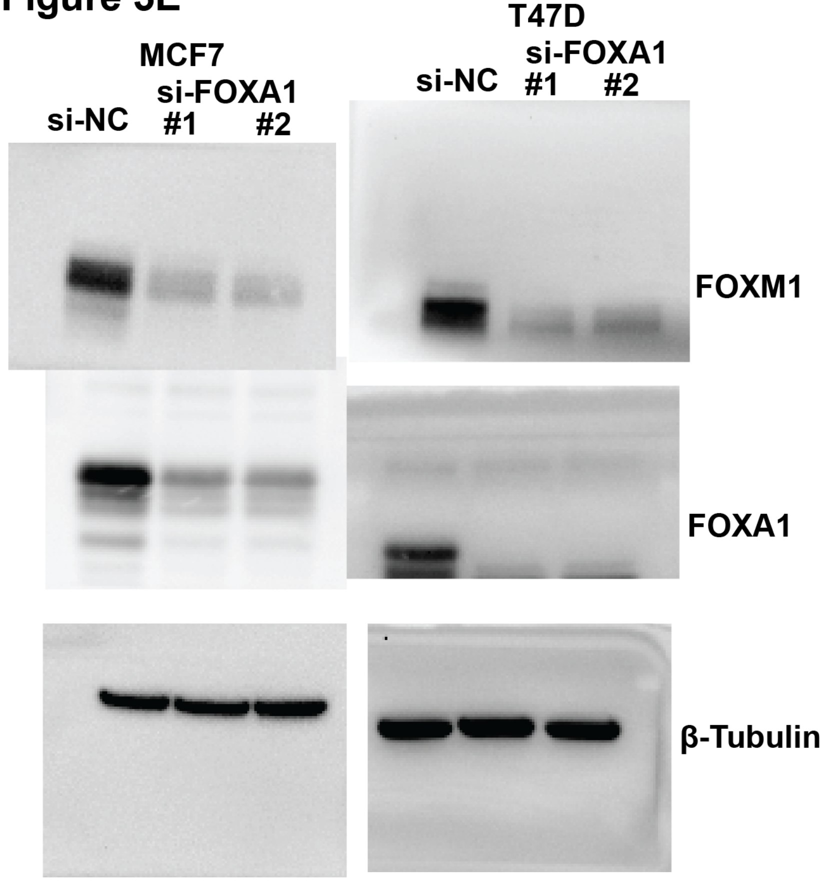

Figure 5H

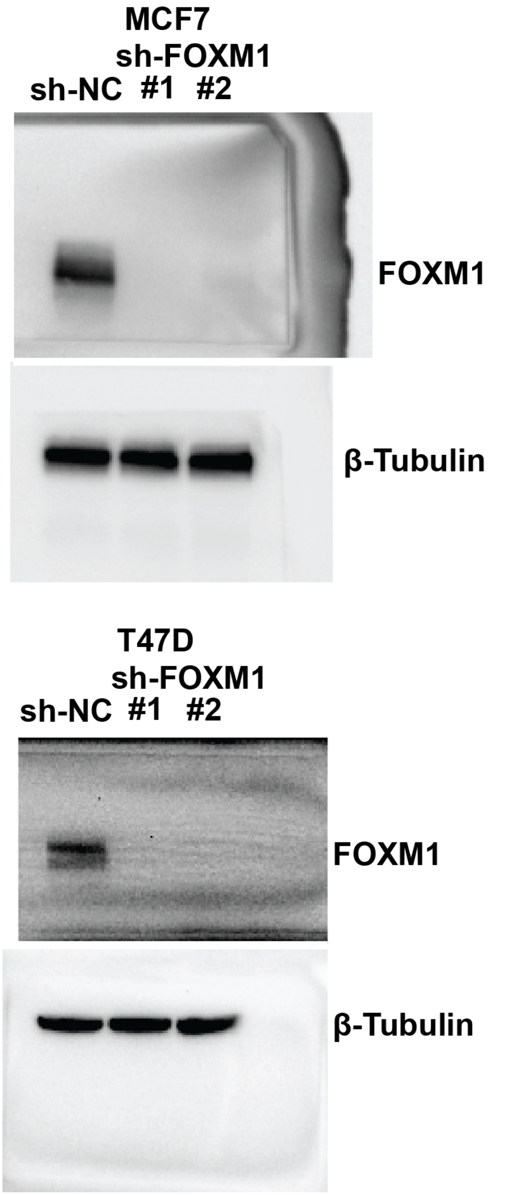

Supplementary Figure 7. The source data of gel pictures in Supplementary Figure 3E, 3H, 4E and 4F.

Supplementary Figure 3E

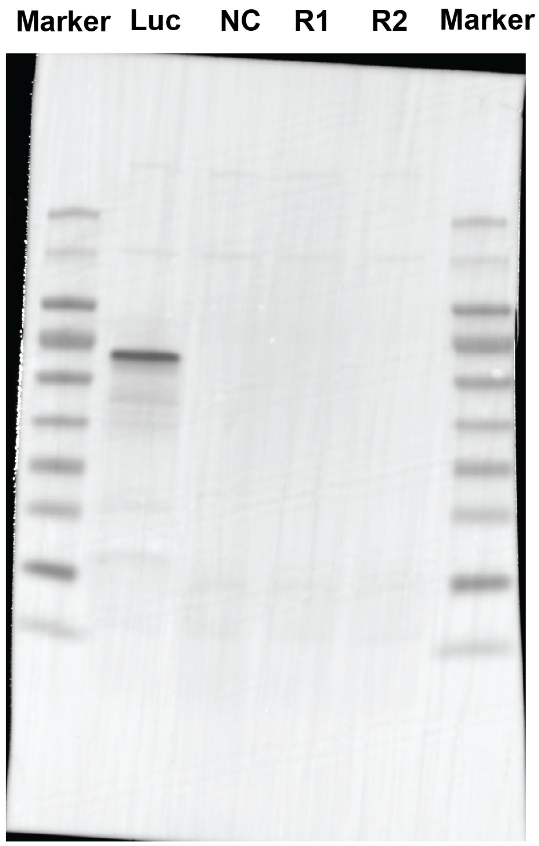

Supplementary Figure 3H

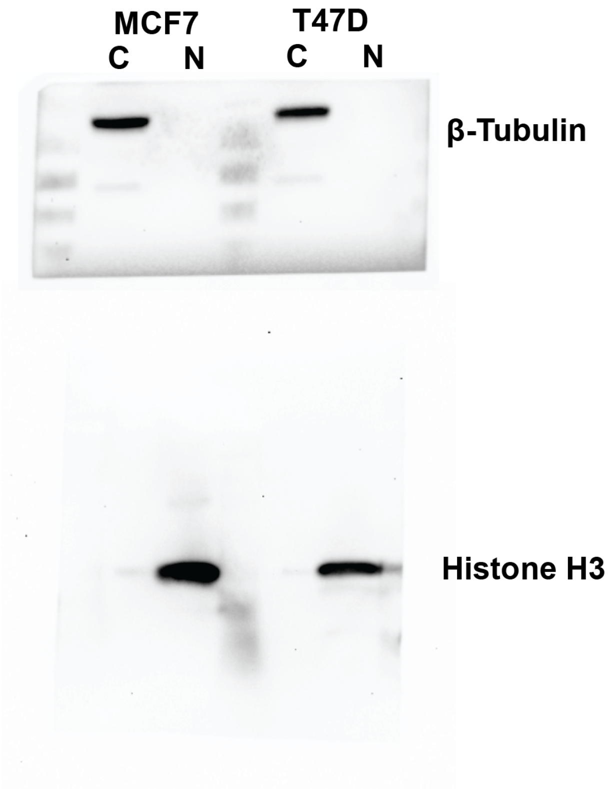

Supplementary Figure 4E

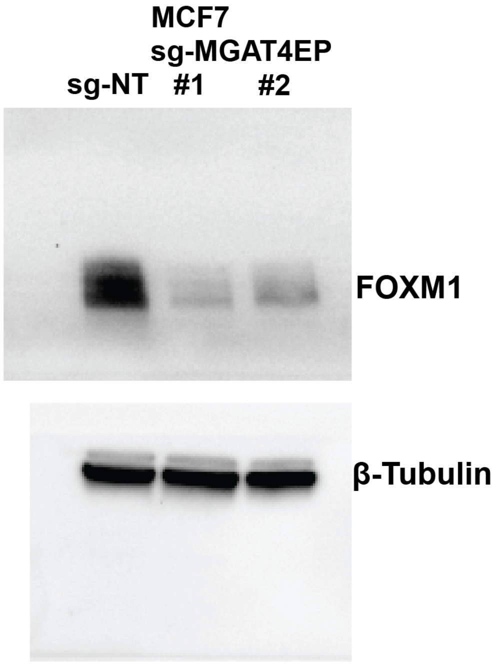

Supplementary Figure 4F

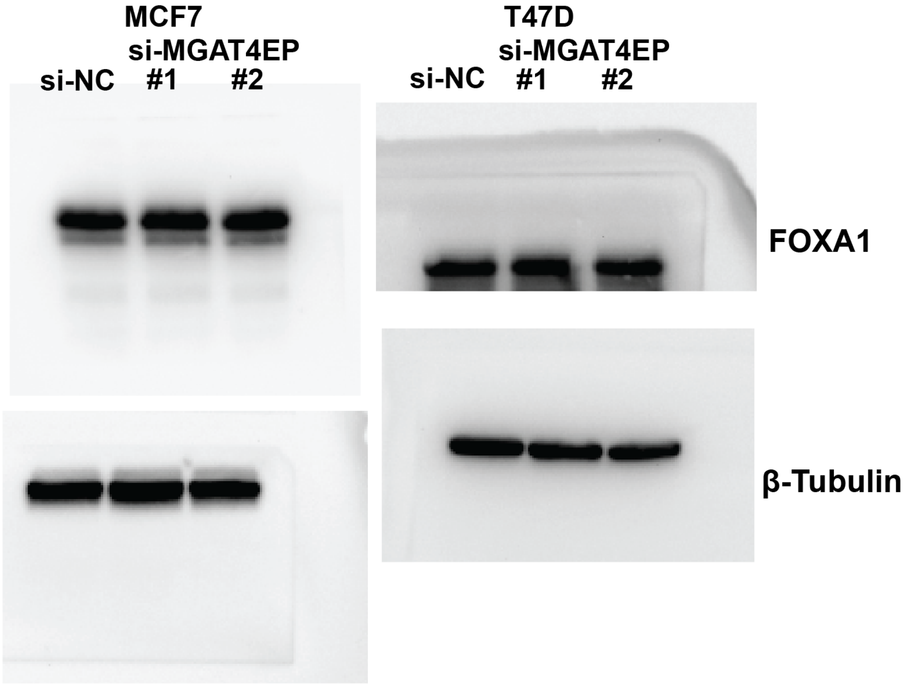

Supplement: Supplementary file 2 — Additional file 2: Supplementary Figure 1-7. [file 13059_2021_2464_MOESM2_ESM.pdf]
